# Strabismus

**Published:** 1850-08

**Authors:** 


*Strabismus.—Division of the Rectus by means of Lane's Knife made by Savigny.*—Every improvement in surgery is interesting, and we eagerly seized the opportunity afforded us by the kindness of Mr. Gay of seeing this instrument used. It is a small curved bistoury, with a partially blunt point. The patient was a little girl. Placed under the influence of chloroform, Mr. Gay, having fixed the eye, introduced the knife by the under side of the rectus, and, holding it flat, passed it vertically on. Owing to its peculiar construction, it went close under the tendon, the point becoming prominent on the other side. On this the operator placed his finger, turning the knife up, when it cut its way out. A second touch of the knife was required, whereupon the globe of the eye instantly resumed its normal position. As nothing can be more simple than this instrument, we sincerely wish to see it tried still further. Its advantages, we are given to understand, are, that from its construction, its point will pass through all the textures external to the sclerotic, but that no force can make it penetrate this membrane.—*London Med. Times.*

